# Latent Autoimmune Diabetes of the Adult (LADA) in a Brazilian Indian

**DOI:** 10.1590/S1516-31802001000200009

**Published:** 2001-03-02

**Authors:** João Paulo Botelho Vieira, Regina Célia Santiago Moisés, João Roberto de Sá, Antonio Roberto Chacra, Sergio Atala Dib

**Keywords:** Diabetes Mellitus, Autoantibodies, Latent Autoimmune Diabetes of the Adult, Brazilian, Indian, Diabetes Autoimune Latente do Adulto, Índio, Brasileiro

## Abstract

**CONTEXT:**

Latent autoimmune diabetes of the adult (LADA) as originally described represents perhaps as many as 10 – 20% of adult-onset patients with diabetes.

**DESIGN:**

case report.

**CASE REPORT:**

A 38-year-old Brazilian Xavante-Jê Indian with Latent Autoimmune Diabetes of the Adult (LADA) is described, coming from the Sangradouro community in Poxoréu, Mato Grosso. The onset of diabetes after reaching 25 years of age, the evolution to insulin deficiency after a period of insulinindependence and the presence of auto-antibodies to glutamic acid decarboxylase (GAD) characteristic of LADA were present. This patient may represent the first case of LADA in a Brazilian with full Indian heritage. Further studies are necessary to verify the prevalence of this new type of diabetes in this population that does not have Caucasoid admixture and has a particular environmental background.

## RESUMO

**CONTEXTO:**

O diabetes auto-imune do adulto, como originalmente escrito, representa 10 a 20% dos doentes diabéticos que iniciam a doença.

**TIPO DE ESTUDO:**

Relato de caso.

**RELATO DO CASO:**

Um índio de 38 anos, do grupo Xavante Jê, pertencente a comunidade de Sangradouro, Poxoréu, Mato-Grosso, Brasil, com Diabetes Auto-imune Latente do Adulto (LADA) é descrito. Início do quadro clínico de diabetes após os 25 anos, evolução para deficiência insulínica após um período de insulinoindependência e presença de auto-anticorpos antiGAD circulantes, características do LADA estão presentes. Esse é o primeiro caso de LADA descrito em populações indígenas. A extensão do estudo é necessária para verificar a prevalência desse novo tipo de diabetes nessa população que não possui componente genético caucasóide e apresenta condições ambientais próprias. Essa caracterização é importante para o diagnóstico, tratamento e prognóstico desses indivíduos.

## INTRODUCTION

Latent autoimmune diabetes of the adult (LADA) as originally described represents perhaps as many as 10 – 20% of adult-onset patients with diabetes.^1^ This slow autoimmune form of type 1 diabetes has been reported in the majority of Caucasian populations.^2-4^ As far as we know, there is no report in the literature regarding LADA in a native Indian population from anywhere in the world. ^5^ We here report the first case of LADA in a Brazilian Indian.

We have prospectively evaluated all 15 diagnosed diabetic patients from a Brazilian Indian community composed of 1000 individuals (336 adults). The majority of these 15 patients are obese and all of them exhibited the clinical course of type 2 diabetes.^6^ So far, they have been treated with diet and oral anti-diabetic agents.

## CASE REPORT

We describe the case of a male 38-year-old Xavante-Jê Indian from the western groups of the Sangradouro community in Poxoréu, Mato Grosso, Brazil. About five years ago, he was diagnosed with diabetes symptoms (polyuria, polydipsia, polyphagia and weight loss) with a fasting plasma glucose (FPG) level of 22.2 mmol/L. After being treated with chlorpropamide for a period of 18 months, he started losing weight and presented with worsening of symptoms of diabetes followed by signs of ketosis. Insulin treatment was then initiated.

At this time arterial blood pressure (BP: 110 × 80 mmHg) or lipidemia (total cholesterol: 28.7 mmol/L and triglycerides: 1.16 nmol/L) were normal but he had a previous history of obesity (BMI > 27 Kg/m^2^). Anti-GAD_65_ was 4.1 U/ml (cut-off = 1.00 U/ml) using a radioimmunoassay kit with human recombinant I^125^ GAD_65_ from Kronus^®^ and IA-2 antibodies were 0.01 U/ml (cutoff = 0.47 U/ml) using a radioimmunoassay kit with human recombinant I^125^ IA-2 from Kronus^®^. The fasting C-peptide level was 0.19 nmol/L (Normal range: 0.11 – 1.18 nmol/L). Recently, the fasting plasma glucose level has varied between 5.9 and 7.2 mmol/L with 50 U of NPH insulin a day. Macroproteinuria and diabetic retinopathy have not been detected.

There is a family history of diabetes that includes an obese sibling who has been treated with diet and oral anti-diabetic agent.

## DISCUSSION

As is known, the usual features of LADA patients are: onset of diabetes at > 25 years of age, clinical presentation masquerading as type 2 diabetes, initial control of hyperglycemia with diet and oral anti-diabetic agents, evolution to insulin necessity within months and some features of type 1 diabetes such as low fasting C-peptide and positive anti-GAD antibodies.^7^ Our patient shared all those characteristics (with age > 35 years at onset of diabetes, he had been non-ketotic and non-insulin dependent for 6 months and insulin deficiency was demonstrated by low serum fasting C-peptide).

Anti-GAD antibodies can be detected in about 10% of type 2 diabetic patients.^8^ However this antibody is more frequent (73.7%) in these patients when they present LADA features.^9^ The low prevalence of IA2 antibodies, as compared with anti-GAD, that has been shown in these patients, can help to explain the lack of this antibody in our patient.

In summary, this patient may represent the first case of LADA^3^ in a Brazilian with full Indian heritage. Further studies are necessary to verify the prevalence of this new type of diabetes in this population that does not have Caucasoid genetic admixture and has a particular environmental background.
